# Supplementary material for: Dynalign II: common secondary structure prediction for RNA homologs with domain insertions
Source: Nucleic Acids Res. 2014 Nov 21;42(22):13939–48. doi: 10.1093/nar/gku1172 (PMC4267632; doi:10.1093/nar/gku1172)
Supplement: SUPPLEMENTARY DATA [file supp_gku1172_nar-02021-z-2014-File012.zip › manual/RNA_class/html/design_8cpp.html]

RNAstructure Classes: E:/RNAstructure/RNA\_class/design.cpp File Reference

- Main Page
- Classes
- Files

- File List
- File Members

# E:/RNAstructure/RNA\_class/design.cpp File Reference

`#include "design.h"`  

|  |  |
| --- | --- |
|  |  |
| Defines | |
| #define | DEBUG |
| Variables | |
| const double | EXTERIORCLOSENESS = 0.10 |
| const double | MULTICLOSENESS = 0.5 |
| const int | MINIMUMLENGTH = 6 |
| const int | two = 2 |

---

## Define Documentation

#define DEBUG

---

## Variable Documentation

const double EXTERIORCLOSENESS = 0.10

const int MINIMUMLENGTH = 6

const double MULTICLOSENESS = 0.5

const int two = 2

---

Generated on Mon Apr 23 16:48:03 2012 for RNAstructure Classes by 
 1.5.7.1
